# Field-Dependent Pollution Model under Polluted Environments for Outdoor Polymeric Insulators

**DOI:** 10.3390/polym14030516

**Published:** 2022-01-27

**Authors:** Rizwan Ahmed, Rahisham Abd Rahman, Arshad Jamal, Ali Ahmed Salem, Bander Saman, Kwan Yiew Lau, Sherif S. M. Ghoneim

**Affiliations:** 1 Faculty of Electrical and Electronic Engineering, University Tun Hussein Onn Malaysia, Batu Pahat 86400, Malaysia; Rizwan.ciit016@gmail.com; 2 Interdisciplinary Research Center of Smart Mobility and Logistics (IRC-SML), King Fahd University of Petroleum & Minerals, Dhahran 31261, Saudi Arabia; arshad.jamal@kfupm.edu.sa; 3 Department Institute of High Voltage and High Current, School of Electrical Engineering, Universiti Teknologi Malaysia, Johor Bahru 81310, Malaysia; en.alisalem@gmail.com (A.A.S.); kwanyiew@utm.my (K.Y.L.); 4 Electrical Engineering Department, College of Engineering, Taif University, P. O. Box 11099, Taif 21944, Saudi Arabia; saman@tu.edu.sa

**Keywords:** polymeric insulators, pollution, dry band, leakage current, layer conductance, finite element method, electric field

## Abstract

In-depth understanding of the pollution problems such as dry bands and the polymeric aging process requires better determination of electric field strength and its distribution over the polymeric surface. To determine the electric field distribution over the insulator surface, this research proposes utilizing a novel approach model based on nonlinear electrical characteristics derived from experimental results for polluted polymer insulators. A case study was carried out for a typical 11 kV polymeric insulator to underline the merits of this new modeling approach. The developments of the proposed pollution model and the subsequent computational works are described in detail. The study is divided into two main stages; laboratory measurements and computer simulations. In the first stage, layer conductance tests were carried out to develop nonlinear field-dependent conductivity for the pollution modeling. In the second part, equipotential and electric field distributions along the leakage were computed using the finite element method (FEM). Comparative field studies showed that the simulation using the proposed dynamic pollution model results in more detailed and realistic field profiles around insulators. This may be useful to predict the formation of dry bands and the initiation of electrical discharges on the polymeric surface.

## 1. Introduction

The outdoor insulator is an essential component of electrical power transmission and distribution systems, considering a single insulator failure resulting in catastrophic failure action of the whole power system. During service life, polymeric insulators are continuously exposed to environmental pollution in the form of industrial waste, agricultural pesticides, and natural pollution. Insulators near coastal areas capture pollution from sea wind in the form of salt, whereas contamination in industrial and urban areas is deposited in ashes and dust [1,2,3]. The contaminants become conductive when they absorb moisture from the atmosphere and form a thin conductive layer, resulting in leakage current. Under nominal system voltages, the water vapors evaporate due to the resistive heating to form dry bands. The phenomena of tracking and erosion occur due to the coupling of electric fields with the voltage gradient across the dry bands [4,5]. The arcs are generated, which may elongate to many dry bands, resulting in complete flashover and power outage under favorable conditions.

Polymeric insulators are becoming more popular among electric power companies with overhead distribution and transmission lines. However, their long-term performance and reliability remain uncertain due to their shorter service life experience than the conventional glass and porcelain insulator [6]. Significant research investigations have been carried out through experimental and theoretical approaches to investigate the performance of polymeric insulators [7,8,9,10,11]. Many of them are focused on electric field computation around the insulator. The investigation of the electric field across the insulator surface leads to a better understanding of pollution issues such as aging and rapid degradation. Furthermore, dry band development prediction may be accomplished more precisely [12].

Measuring voltage and electric field distribution around practical insulators is complex, and even more problems arise under polluted surface conditions. Nanoparticles can be used as a filler in polymeric insulators. The ultra-small nanoparticles exhibit a high surface area with narrow pores, which serve as conductive channels for the proficient passage for ions/electrons exchange during electrochemical energy storage reactions [13,14]. The electrostatic probe [15] technique is subjected to errors; however, the errors can be mitigated using complex and advanced electric field detection systems [16]. Numerical techniques are also employed to analyze insulators surrounding electric fields. It can be achieved by employing computational methods such as the finite element method [17,18], boundary element method [19], and charge simulation method [20]. These computation techniques are cost-effective, efficient, and accurate compared to laboratory testing, which requires a sophisticated environment and a complicated testing process. Moreover, advanced numerical packages can solve complex field models with less computation time and increased accuracy.

In literature, the researchers usually assume the single and linear conductivity of the pollution layer while simulating the outdoor insulator [21,22,23,24,25]. This assumption is not always valid in a practical scenario. The tangential component of the field dramatically influences the surface conductivity. Due to surface heating, the conductivity decreases with time as moisture evaporates from the contamination layer [26,27,28,29]. The drying effect of the pollution layer will account for electric field strength. Therefore, it is critical to investigate the nonlinear conductivity behavior on the insulator surface in order to have a better understanding of pollution layer properties such as electric stress (electric field) [30,31]. The proposed nonlinear electrical property is obtained from experimental results of low voltage layer conductance tests. The paper explains the experimental program and describes electric field distribution simulation results on the polymeric surface. A 2D insulator structure with a uniform pollution layer is modeled under a humid atmosphere; fog and light rain are accounted for by dynamic wetting and drying actions with simplified assumptions.

Field profiles obtained using the proposed dynamic model are analyzed and compared with those obtained with a linear conductivity value. The FEM analysis of the nonlinear contamination model is presented, which helps predict dry band formation. Polymeric insulators under normal conditions have excellent hydrophobic properties hence they are rarely subjected to the uniform wetted surface when new or upgraded. This study will improve the understanding of polluted outdoor insulators’ linear and nonlinear effects on electric field distribution.

## 2. Materials and Methods

### 2.1. Experiment Sutep

#### 2.1.1. Basic Insulator Structure

**Figure 1:**
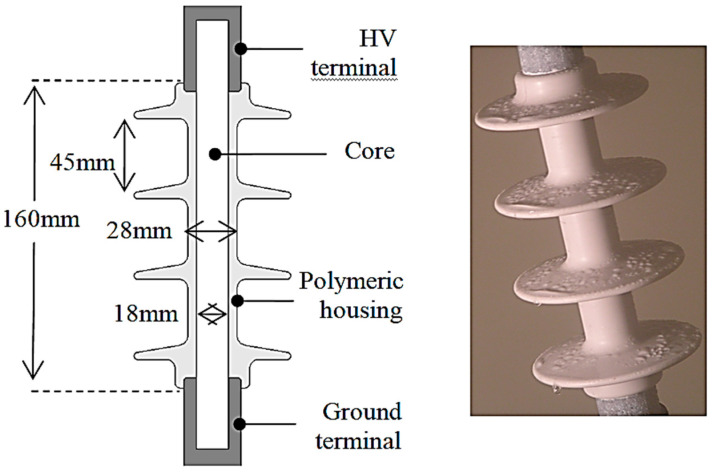
Insulator profile and dimensions.

The profile and dimensions of the test insulator used in this investigation are detailed in Figure 1. It is a four-shed 11 kV overhead line polymeric insulator employed in a light-polluted environment. The basic construction of any polymeric insulator is composed of three constituents, i.e., insulation housing, metal flanges, and a fiber core. The HV and ground terminal were crimped to a fiber-reinforced plastic (FRP) core at a separation distance of 160 mm for the test insulator. Core and metal fitting provided supplementary mechanical support to an overhead transmission line. The insulation housing was silicon rubber (SiR), a synthetic composite compound with excellent hydrophobic properties. The water on the insulator surface remained as droplets; hence the risk of dry band formation and leakage current was minimized. The synthetic composite was used to protect the core and provide electrical insulation under wet conditions. The measured leakage path on the polymeric surface was approximately 360 mm.

#### 2.1.2. Laboratory Test Setup

The measurements were performed using a low voltage test setup based on the procedures outlined in the IEC-507 Standard. However, a non-standard test method was adopted to investigate the drying of wet pollution surfaces caused by joule heating.

**Figure 2:**
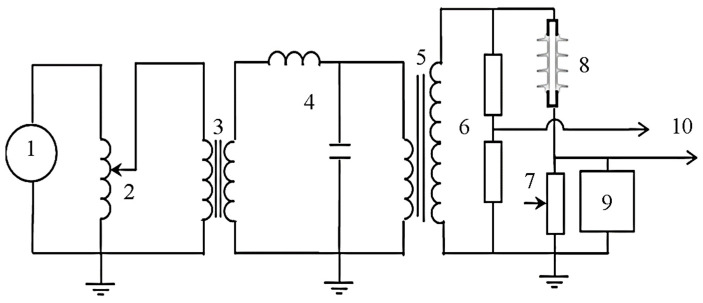
Circuit diagram of the experimental setup. 1: 240 V AC, 2: Voltage regulator, 3: 1:1-Isolation transformer, 4: Low pass filter, 5: Step-up transformer, 6: Voltage divider, 7: Variable resistor, 8: Test I, nsulator, 9: Protection box, 10: Digital oscilloscope.

Figure 2 show the schematic diagram of the test circuit used for the low voltage conductance measurements. The applied voltage was supplied and controlled by a voltage regulator (1) that was connected to a step-up 7.5 kVA transformer (5) through an isolation transformer (3) and low-pass filter (4). The 1:1 isolation transformer prevents differential currents in the earthing circuit, other than to protect in the event of transients. The inductor-capacitor (IC) filter was introduced to suppress high-frequency noise from the main supply; it ensured an improvement in signal quality for the test insulator on the visual probe. The potential difference across the insulator was obtained at the low voltage arm of resistive divider (6), rated at ratio 2000:1. At the same time, the leakage current was measured through a variable shunt resistor (7) located in series with an insulator (8). A digital storage oscilloscope (DSO) (10) was used to record and display both leakage current and voltage. A circuitry box consisting of gas discharge tube zinc oxide varistors and transient suppressors were connected in parallel to the shunt resistor for protection overvoltage at the data acquisition device.

Results from the layer conductance test were employed to symbolize the properties of the contamination layer to model in the later simulation work.

#### 2.1.3. Preparation of Artificial Contaminant

A solid layer method was used to uniformly contaminate the test insulator using kaolin suspension. The contamination slurry was produced by mixing kaolin weighting 40 g in one liter of demineralized water. Following the IEC-507 Standard criterion for heavy pollution, sodium chloride (NaCl) was added to obtain the volume conductivity of 11.2 S/m at 20 °C. TRITON-X 100, a nonionic wetting agent at 0.05% concentration, was also added to improve the wetting mechanism allowing a uniform contamination layer on the insulator surface. Test insulators were thoroughly cleaned using detergent and water prior to applying contamination. The contamination slurry was flooded over the insulator surface using the ‘flow on’ technique. The contaminated insulators were placed at room temperature for eight hours to dry out prior to experimental validation. If pollution was not uniformly distributed over the insulator surface, the insulator was re-polluted until the desired level of uniformity was achieved.

#### 2.1.4. Wetting

Test insulators were artificially polluted following standards by spraying fog particles over their surface. While using the solid layer method, there was a concern about the washing effect. The contamination layer may gradually wash off the insulator surface by employing constant fog generation. This research focused on the effect of the drying process (Joule heating) on surface conductance. By employing non-standard wetting, the desired results may not be achieved successfully. As for an alternative, the test polluted insulator was uniformly wetted using the ‘flow-on’ technique by immersing it in water, similar to the contamination suspension applying technique. Special attention was taken to analyze the contamination coating attached during the dipping process on the insulator surface. The test insulator was assumed to be at its peak conductance level when the contamination surface was entirely wet, demonstrating the most severe operating condition in practice. With this wetting technique, the water deposition on the insulator surface can be controlled efficiently without the washing effect and time to reach maximum conductance while using fog. The two types of wetting considered were fog and light rain.

In fog (uniform wetting), tiny water particles move in a foggy atmosphere in a languid and random motion. The water droplets can access insulator surface in all directions. For this reason, it is assumed that under fog conditions the wetting action is uniform and constant on outdoor insulator surface.

In contrast, in light rain (non-uniform wetting), the dissipated power and wetting rate, i.e., rate of moisture deposited on outdoor insulators, are the properties that govern the dry band formation. The condition for dry band formation is when the drying rate is greater or equal to the the wetting rate. In heavy rain case, the surface discharges and drybands are less significant. The rain could wash out the pollutants and re-wet the dry regions on the insulator surface, reducing the probability of electrical discharges. The problem arises when polluted insulators are exposed to drizzle or light rain weather conditions.

**Figure 3:**
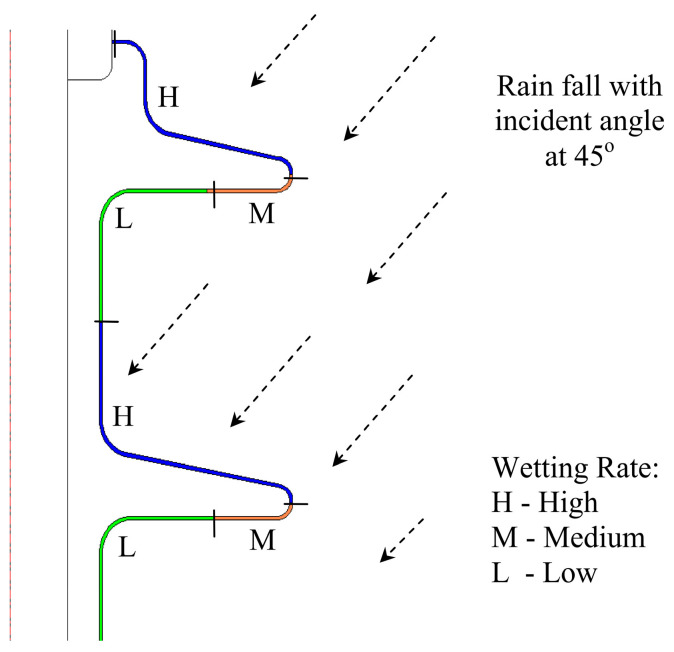
Subdivision of pollution layer under light rain conditions.

Unlike fog particles, water from the rain may not reach the entire polymeric surface uniformly. Surfaces that are exposed have a high wetting rate as compared to under-shed regions. In Figure 3, the contamination layer is divided into three parts that are H (high), M (medium), and L (low) for modeling purposes. The regions are categorized based on the actual wetting process in nature. The H region is directly exposed to the atmosphere; hence, rain droplets are deposited more in that region. The half under-shed region M is moderately exposed to rain droplets, and region L is the low wetted region located under the insulator shed.

#### 2.1.5. Test Procedures

The layer conductance test was performed using an existing fog chamber test facility in Cardiff University. Pre-wetted polluted insulators were vertically suspended in a fog chamber for voltage energization. Instead of employing a standard intermittent voltage application, a non-standard low voltage test procedure was adopted. A constant voltage energization, starting with 300 V, was applied continuously during the test period. The resulting leakage current, *LC*, was recorded at every one-minute time interval, and the first reading at t = 0 min was taken at the point of voltage energization. Ac waveforms, leakage current measurements, and applied voltage were stored and displayed using a digital oscilloscope. The test stops when the leakage current becomes negligible and peaks due to electrical discharge dominating the current waveform. Similar test procedures were repeated on other test insulators, each with different energization voltages, V_E_ of 600 V, 900 V, 1.2 kV, and 1.5 kV.

### 2.2. Development of Nonlinear Pollution Model

#### 2.2.1. Dry Bands

Pollution deposition on the surface of energized outdoor insulator experiences negligible risk of capacitive current under dry conditions due to high surface resistance. However, under wet conditions, the insulator resistance drops significantly, resulting in leakage current flow and increased current density along the creepage path from the HV terminal to the GND terminal. The electric field *E_S_*, and longitudinal current density *J_S_*, are always non-uniform due to the insulator sheds and Shank’s structure. The electric field of a contamination layer with resistivity $\rho_{s}$, can be expressed as [32]:

(1) $$E_{s}=\rho_{s}J_{s}=\frac{J_{s}}{\sigma_{r}}$$

The current density varies with insulator geometry at Shank regions where the circular surface is primarily minor, and the current density is maximum. An increase in electric field and high current density results in increased power dissipation, becoming the energy source for heating and dry band formation. The equation for the power dissipation *P_E_* for a contamination layer is represented as follows:

(2) $$P_{E}=E_{s}J_{s}=\frac{J_{S^{2}}}{\sigma_{r}}$$

#### 2.2.2. Surface Conductance

**Figure 4:**
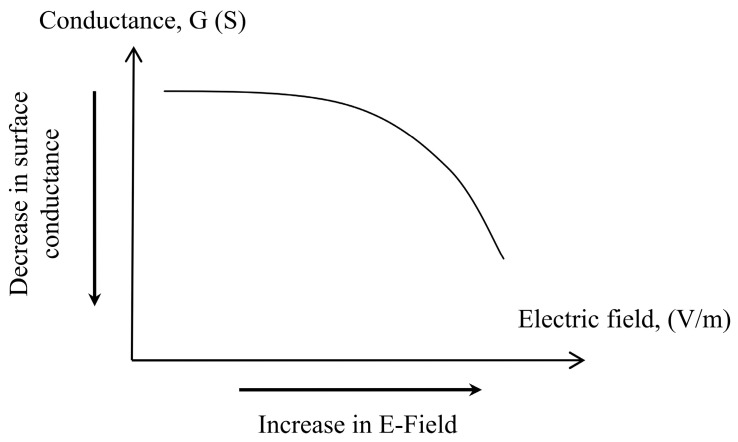
General representation of surface conductance as a function of the surface electric field.

The leading cause of leakage current is the moisture content presented in the outdoor insulator pollution layer. The value of leakage current increases with an increase in moisture level [33]. Surface conductance maximum value is achieved when the contamination layer is perfectly saturated with moisture. Surface conductance maximum value is expected to decrease as moisture evaporates from the contamination layer due to the Joule heating effect. The evaporation rate of water content is directly proportional to the electric field, as stated in Equation (2). Hence, surface conductance changes have a minimal value in the low field region and a maximum value in the high electric field region. When the electric field reaches the breakdown threshold, it drops abruptly. Experimentally, the breakdown was measured to be approximately 10 kV/cm. Exceeding this point, the pollution layer was considered dry, imposing a highly resistive region on the insulator surface. Figure 4 below represent the general graphical representation of the field conductance relationship. Nevertheless, this dependency was determined and confirmed experimentally in the low voltage layer conductance tests.

The leakage current measurements from this experiment were used to obtain the contamination layer conductance obeying the following Equation:

(3) $$G_{LC}=F\times \frac{I_{LC}}{V_{C}}\;$$

where $I_{LC}$ is the leakage current (mA), *Vc* is critical voltage (kV), and *F* is the geometrical form factor of the insulator, which can be determined from the insulator profile using Equation:

(4) $$F=\overset{L}{\underset{0}{\int }}\frac{l}{2\pi r\left(s\right)}ds\;$$

The term $2\pi r\left(s\right)$ in Equation (4) represents the insulator surface circumference at distance *l* along the creepage path, *L*.

To ensure the consistency of pollution level on each test insulator, a precondition measurement is performed prior to commencing the low voltage test. The insulator is energized with a relatively low voltage (150 V) that is adequate to establish a measurable leakage current. It is applied only for a short while to avoid the possibility of surface heating and evaporations. The computed conductance with 10% tolerance is used to reference the pollution level on each insulator surface. If the conductance value falls outside the acceptance criterion, the insulator is washed off and polluted again to achieve the desired conductance level. A deviated value suggests that the insulator is not well polluted with the presence of a patchy or unpolluted surface.

### 2.3. Simulation of Polluted Insulator

#### 2.3.1. Material Properties

The essential characteristics analyzed in this paper are relative conductivity and permittivity. The relative conductivity and permittivity of the materials used to make up each portion of the insulator depicted in Figure 1 were entered into the COMSOL Multiphysics software (5.5, COMSOL Multiphysics^®^, Stockholm, Sweden). Air, insulator terminals, core, and silicon rubber have relative permittivity values of 1, 1, 7.1, and 4.3, respectively. The conductivity of air, core, and silicon rubber is 1.0 × 10^13^ (S/m), while the conductivity of insulator electrodes is 5.9 × 10^7^ (S/m) [12]. The deposition of pollution on outdoor insulators is non-uniform and strongly dependent on the location and nature of the environment. To reduce modeling complexity, the contamination in this work is uniform at 0.5 mm thickness over the insulator surface. The conductivity is specified as a function of an electric field, *σ*_p_ = *f*(*E_S_*), as described earlier in Section 5. When the pollution is in a conduction state, water is considered a dominant factor; hence the relative permittivity value is assumed as 80. The HV top terminal of an insulator is fed with an AC voltage of 18 kV, and the bottom terminal is grounded. Under light-polluted conditions, this voltage indicates phase to earth maximum voltage for a typical 11 kV system described in the standards [34].

#### 2.3.2. Finite Element Method (FEM)

**Figure 5:**
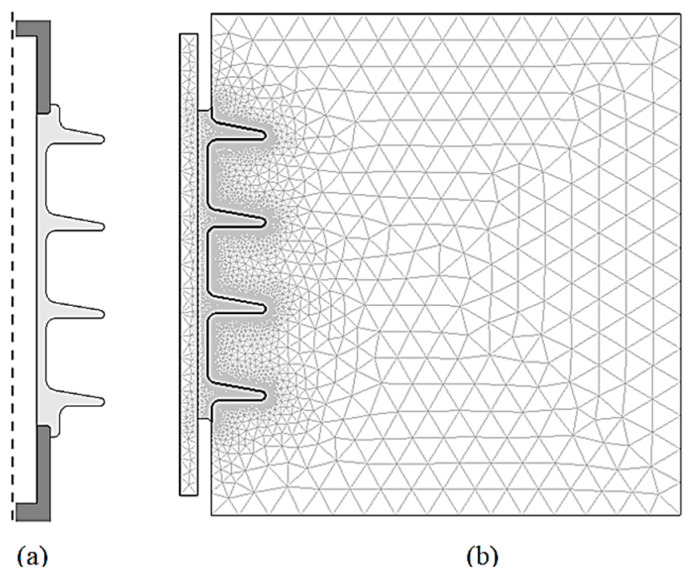
(**a**) A 2D symmetrical insulator model and (**b**) Mesh discretization of the domain problem.

Field computations are executed by means of a commercial FEM package, COMSOL Multiphysics. A 2D insulator geometry modeled in this simulation is shown in Figure 5a, where only half of the insulator structure is considered due to its symmetrical property. Figure 5b show the mesh elements of the problem domain, with additional refinement in the leakage path. The insulator model is evaluated using the ‘Quasi-Static Electric Current’ module in the time-steps domain solver. This module takes on gradually varying electromagnetic fields and currents, suitable for HV applications and insulator issues operation at power frequency [35]. The execution mode requires the user to specify the permittivity and conductivity of the pollution model specified by the nonlinear expression for field analysis and computations [36].

## 3. Results and Discussion

### 3.1. Experimental Results

**Figure 6:**
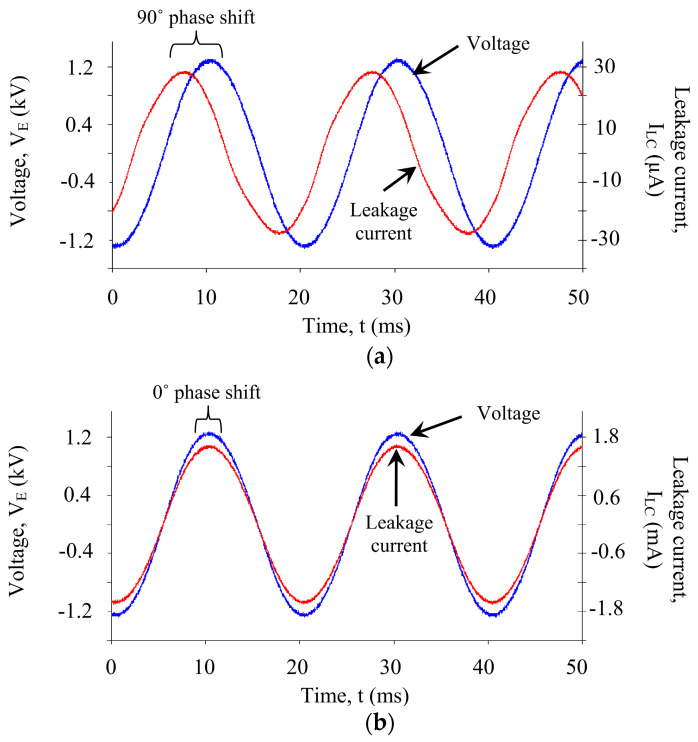
Voltage and leakage current of an insulator under test: (**a**) clean condition; (**b**) Polluted condition.

The AC waveform examples of leakage current and applied voltages from low voltage conductance tests are shown in Figure 6. The traces are recorded at the point of voltage energization (900 V) for the insulator under wet and dry surface conditions. Under dry surface conditions, the magnitude of the leakage current is small, predominantly capacitive, with a phase shift of 90°. The current magnitude increases from 0.02 mA to 1.2 mA, and the phase difference is zero, indicating the resistive current conduction. However, both the phase shift and magnitude of leakage current change under wet conditions, as shown in Figure 6.

The reference leakage current and the computed surface conductance from the precondition measurement with voltage energization 150 V are tabulated in Table 1. The minor discrepancies between insulators give a positive indication that a uniform pollution level on each test insulator is successfully achieved.

**Figure 7:**
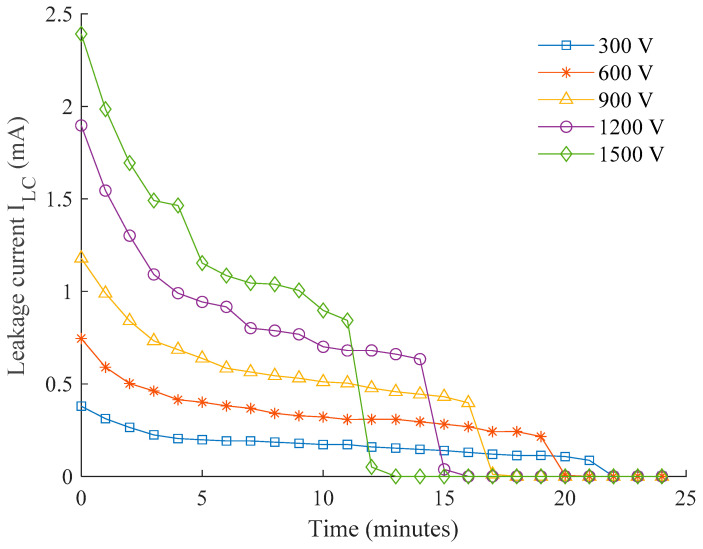
The plot of leakage current for different energization voltages during a period of testing.

**Figure 8:**
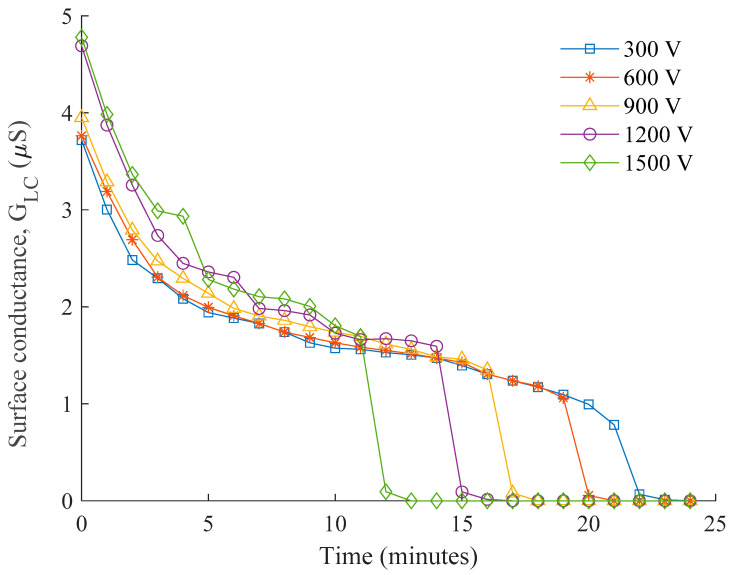
The plot of surface conductance for different energization voltages during the testing period.

The experimental results of leakage current measurements and the corresponding layer conductance are plotted in Figure 7 and Figure 8. From these figures, it is identified that the most significant current conduction occurs at the point of voltage energization (t = 0 min). The pollution layer at this instant is subjected to a high moisture level after being dipped in water, thereby imposing a maximum layer conductance, as depicted in Figure 8. The slight variations in the conductance between 3.8 µS and 4.8 µS can be the evidence for consistency of the pollution level achieved on each insulator.

As shown in Figure 7 and Figure 8, the current flowing through the conductive pollution film gradually decreases with time, shown by the reduction trend in both leakage current and conductance curve. This indicates surface evaporation due to Joule heating during the period of voltage energization. The steep gradient at the beginning of voltage applications suggests an accelerated evaporation process that facilitates the drying out of the wet contamination layer. As a decrement in moisture level in the contamination layer occurs, the resulting leakage current has low magnitude, and there is inadequate heat energy to cause a further reduction. Hence, slight changes in leakage current and conductance are observed over a longer period of time.

The surface conductivity rapidly falls towards the end due to the formation of dry bands on the outdoor insulator surface. The time taken for this rapid fall is solely dependent on variation in the applied voltage. Higher voltages that generate greater heating energy as expected require a shorter period to cause a dry band, and similarly, lower voltage requires longer, as clearly shown in the plots. A series of sudden changes in the leakage current and conductance are found at the 1.2 kV and 1.5 kV plots, suggesting the presence of discontinuous conduction when the intermittent dry bands occur. From naked observation, severe electrical discharges are rarely seen on most of the ac waveforms except a couple of apparent spikes for the insulators energized with 1.2 kV and 1.5 kV.

### 3.2. Derivation of Nonlinear Field Dependent Conductivity

The effective overall electric field, *E*, along the creepage path at a total distance, *d_C_*, for an insulator with energization voltage, *V_E_*, can be determined using:

(5) $$E=\frac{V_{E}}{d_{C}}\;$$

For a conductive pollution film on the insulator surface, the change in conductance, ∆*G_LC_*, is given by the difference between conductance values measured at two different times. Supposing negligible conductance at the time when a dry band occurs, ∆*G_LC_* approaches maximum conductance level under wet surface conditions for a complete drying process, as shown in Equation (6).

(6) $$\Delta G_{LC}=G_{LC\;min}-0=G_{LC\;max}$$

If the wet pollution surface requires *t* minutes to form a dry band, the rate of change in surface conductance, *R*_∆*G*_, can be expressed by

(7) $$R_{\Delta G}=\frac{\Delta G_{LC}}{t}$$

**Figure 9:**
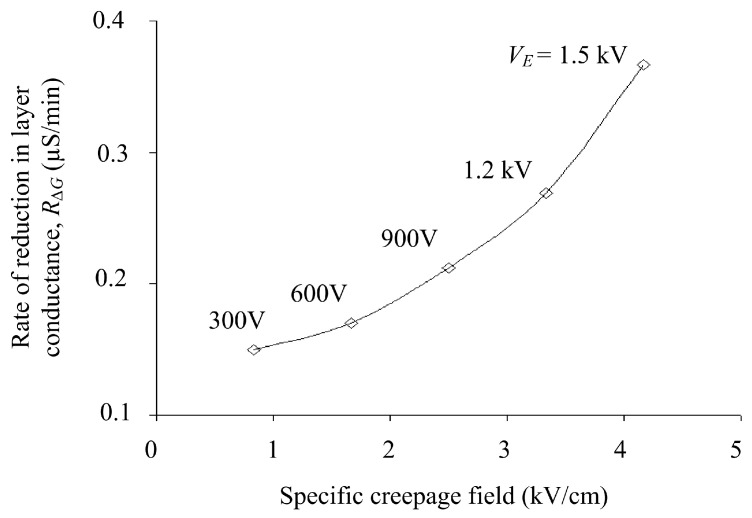
Rate of change in surface conductance as a function of specific electric field on polluted insulator energized with different voltage levels.

Figure 9 show *R*_∆*G*_’s plot as a function of the specific creepage electric field during the drying process. The rate of change of conductance in layers related to evaporation rate also increases with the increase in an electric field. The high magnitude of the electric field produces enough heat energy, which results in an accelerated drying process and hence, results in a higher reduction rate in surface conductance.

**Figure 10:**
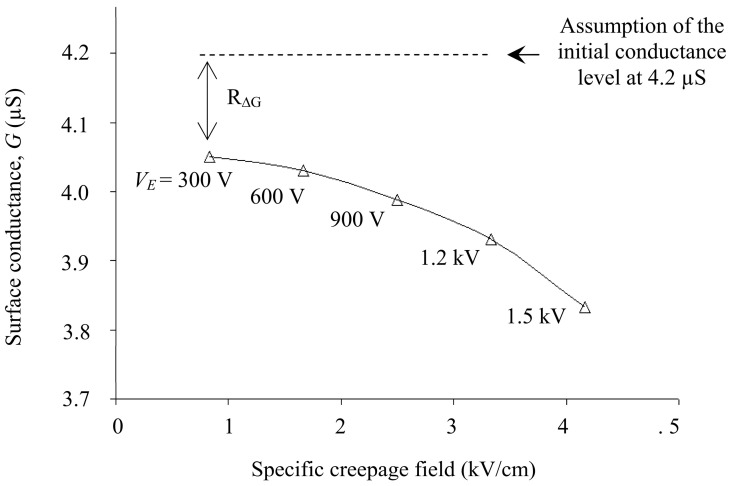
Surface conductance as a function of specific electric field on polluted insulator energized with different voltage levels.

For modeling and simulation purposes, the relationship in Figure 4 is transformed into a field-conductance plot, shown in Figure 10. It is observed that surface conductance has an inverse relationship with an electric field. Surface conductance is considered maximum at 4.2 µS when the polluted insulator is wet. This is the average maximum conductance measured at t = 0 min (see Figure 8) in the previous low voltage test. The maximum surface conductance is expected to decrease by *R*_∆*G*_ due to the evaporation and drying effect when subjected to an increase in an electric field. However, the relationship at a higher field level could not be drawn due to insufficient experimental data at higher energization voltages. There are severe electrical discharges when the test insulator is energized with voltage greater than 1.5 kV, which affects the experimental results presented on the oscilloscope. As a solution, the plot extrapolation method anticipates characteristics over a broader range of electric fields.

**Figure 11:**
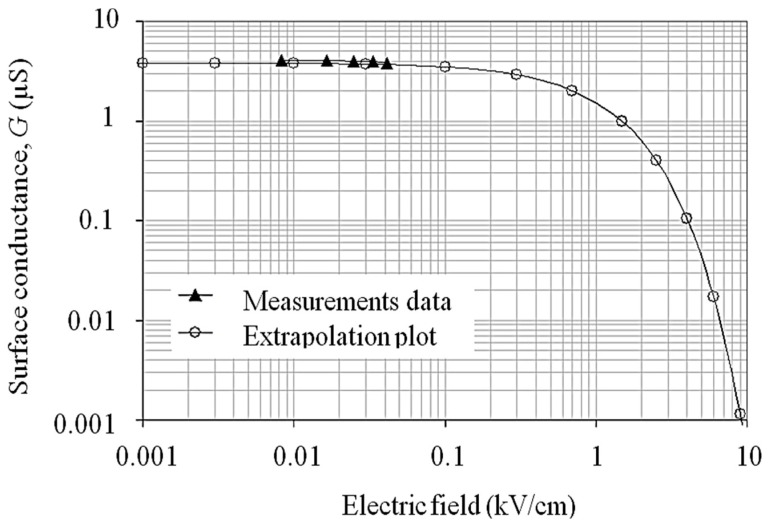
Plot extrapolation of surface conductance for a wider electric field range in a log–log graph scale.

Figure 11 represent the extrapolation plot of the field-conductance relationship for higher field approaching. The breakdown threshold at 10 kV/cm was developed using a curve fitting tool in MS Excel. The trend clearly shows an exponential decay in the log–log plot. The relationship between layer conductance, *G*, and electric field, *E*, can be approximated by:

(8) $$G=4.2\times 10^{-6}e^{-9\times 10^{-5}\left(E\right)}$$

### 3.3. Pollution Model Results under Wet Weather Conditions

In this section, fog and light rain wet effects on surface conductance were discussed.

#### 3.3.1. Pollution Model Results under Fog

**Figure 12:**
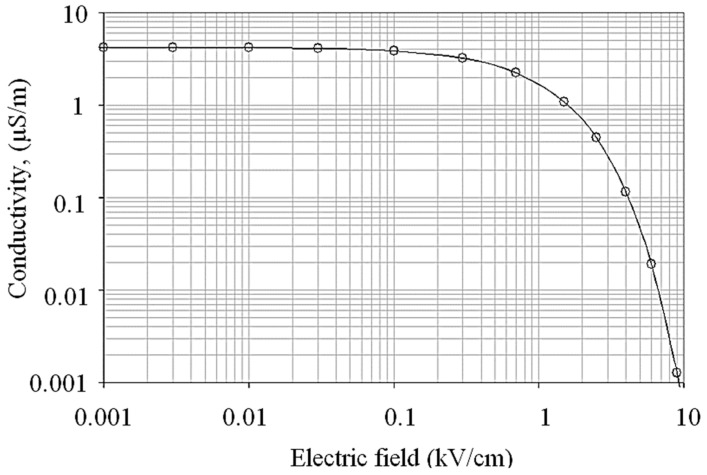
Field-conductivity relationship for the pollution model under fog weather conditions (i.e., uniform wetting action).

Figure 12 present a graph of pollution conductivity as a function of the electric field proposed for simulation under fog conditions. It is similar to the extrapolated curve obtained in Figure 9.

At a lower magnitude of an electric field, the conductivity value is maximum and nearly constant. The contamination layer is fully saturated with moisture and dissipated energy, causing negligible heat due to the low strength of the electric field. The conductivity decreases steadily, indicating an effect of water evaporation as the electric field rises. When it exceeds a specific field threshold, the subsequent drying action due to excessive surface heating triggers a quick action of conductivity reduction, indicated by the field region above 1.0 kV/cm in Figure 10. Due to the drying effect, the field magnitudes higher than 10 kV/cm and negligible electric surface conductivity occurs, altering the conductive wet region into dry and highly resistive regions on the outdoor polymeric insulator surface.

#### 3.3.2. Pollution Model Results under Light Rain

**Figure 13:**
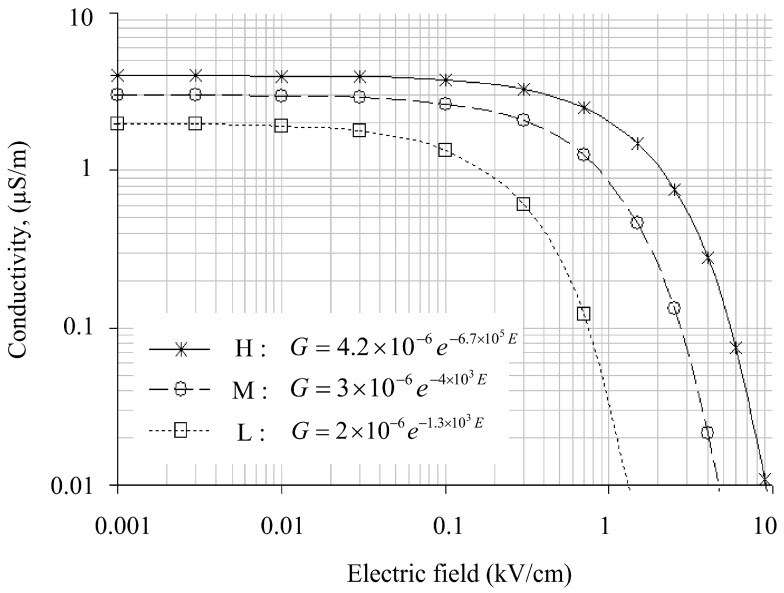
Field-conductivity relationship for the pollution model under light rain weather conditions (non-uniform wetting action).

The independent surface conductivity graph of contamination layers under light rain conditions is shown in Figure 13. H, M, and L curves are marked based on the contamination model in regions H, M, and L. The curves have a big difference in field threshold and initial conductivity, as can be observed. The wetting under light conditions of outdoor polymeric insulators causes these differences. Curve H has the highest electric surface conductivity of 4.2 µS/m as it was more exposed and highly saturated with moisture. The M and L curves show slightly lower conductivity values of 3.0 µS/m and 2.0 µS/m compared to moderate and low moisture regions. The threshold governs the field value at which the contamination layer conductivity swiftly decreases. The areas with low field threshold values demonstrate less wetted regions; hence, these regions are subjected to a high probability of dry band formation. Therefore, the pollution model presented for region L shows the lowest field threshold value, trailed by the M region’s moderate value. The H region faces the highest field threshold value.

According to the comparison of fog and light rain curves, surface conductance and electric field are lower in light rain than in fog conditions. This is because rain may wash away contamination on insulator surfaces. Furthermore, depending on the fall angle and wind direction, some areas of the insulator, such as region L, do not become wet during rainfall, whereas the insulator was wet completely under the fog.

## 4. Simulation Results

The electric and potential field distribution over the surface of the polymeric insulator is analyzed and computed. The simulations are performed for dry and wet contamination surfaces with a conductivity of 4.2 µS/m. These are the common modeling conditions presented in the majority of literature published where the effects of drying and wetting processes are not considered. The simulation results will be implemented as a tool for comparison and control.

**Figure 14:**
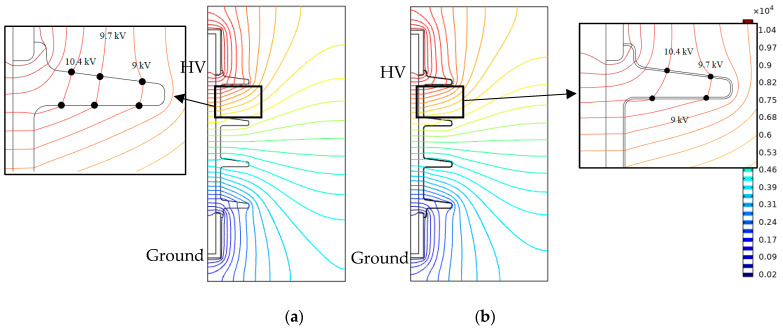
Equipotential around insulator under (**a**) dry clean surface and (**b**) uniformly polluted condition with a single conductivity of 4.2 µS/m.

**Figure 15:**
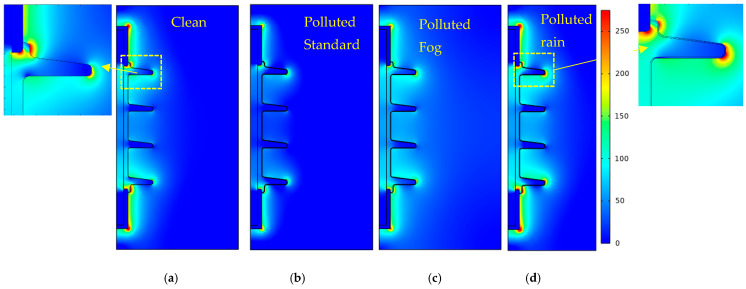
2D Electric field distribution of insulators for: (**a**) Dry clean; (**b**) polluted (standard model); (**c**) Polluted (nonlinear model (fog)); (**d**) Polluted (nonlinear model (rain)).

The equipotential electric field profiles for uniformly polluted and dry clean surfaces are shown in Figure 14. The equipotential lines under polluted conditions are uniformly stretched over the insulator surface. Under a clean, dry surface, the field lines are concentrated in regions near metal fitting due to high field strength (Figure 15). The corresponding electric field distributions on the insulator surface under clean dry, polluted (standard model), polluted (nonlinear model (fog)), and polluted (nonlinear model (rain)) conditions are compared in Figure 15. This field component drives the leakage current on the surface of the polymeric insulator.

**Figure 16:**
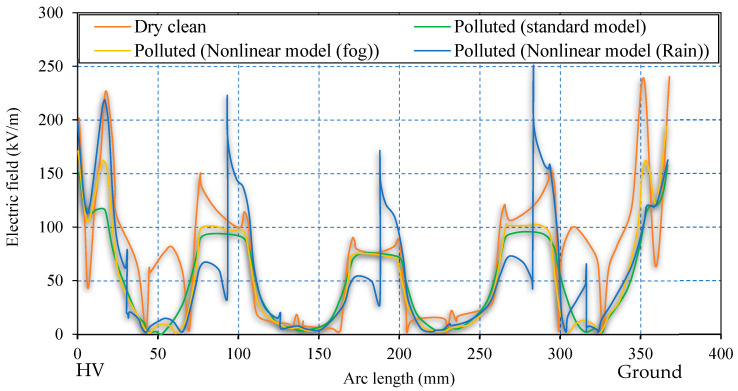
Comparison of tangential electric field distribution for insulator under dry–clean and polluted surface conditions (standard model (single conductivity of 0.6 µS/m (green line)), nonlinear model (fog) (orange line), and nonlinear model (rain) (blue line)).

Figure 16 reveal the proposed models’ electric field distribution compared to the clean condition. The contamination models, as can be seen from Figure 10, are characterized by nonlinear field-dependent conductivity. Both the field profiles show a slight difference. The redistribution of an electric field is indicated by the dynamic model. The terminals are subject to local stress raises from the standard value of 120 kV/m to 160 kV/m, revealing 33% enhancement in the field. A moderate rise in the electric field, about 10%, is observed in the shank regions 1 and 3. These variations occur because of surface conductivity reduction at higher field regions due to the drying threshold. The results interpreted the act as an acceleration process for dry band formation as redistribution results in higher field values following the heating effect.

Assuming the wetting was non-uniform, in Section 3.3, the properties of the pollution model are presented, which were also taken for the simulation model. The electric field distribution of nonlinear pollution simulation model offers a series of peaks on the different surfaces of the outdoor polymeric insulator. Analogous to the fog conditions, this series of peaks can be minimized by reducing the conductivity of pollution due to the drying effect reflected in the current investigation. It can be analyzed from Figure 3 that regions under shelter are less wetted regions L that increase field strength. Stress on the sheltered region near the ground terminal increases noticeably by 83.8% from 120 kV/m up to 250 kV/m. Similarly, significant field increase is also spotted on the Shank’s regions, indicating an area vulnerable to electric discharge activities. The upper shed surfaces, represented by region H (see Figure 11), including the one closest to the HV terminal, on the other hand, show a favorable change in the electric stress. This could be due to the regions that are exposed to the high wetting action. Electrical discharges and dry bands are commonly established in shank regions, as depicted in most experimental work, which correlates well with the proposed model’s simulation results.

## 5. Conclusions

A pollution model with nonlinear field-dependent conductivity is proposed to compute the electric field distribution along the leakage path of outdoor polymeric insulators. An experimental procedure including a low voltage layer conductance test has been carried out to obtain a surface conductance curve as a function of a specific creepage field. The measured breakdown voltage threshold was around 10 kV/cm under uniform wetting. The nonlinear electrical properties of the pollution to be used in FEM modeling are derived from the extrapolation plot of the surface conductance curve. As for the dynamic aspects, wetting and drying are taken into account and described with simplified assumptions to characterize the pollution under fog and light rain weather conditions.

The field-dependent model revealed distribution with a series of peaks at different locations on the polymeric surface. The terminals were subject to local stress raises from the standard value of 1.2 kV/cm to 1.6 kV/cm, indicating about 33% field enhancement. A moderate rise in the electric field, about 10%, was observed in the shank regions 1 and 3, primarily due to the reduction in surface conductivity when reaching the drying threshold at higher field regions.

By combining the field-dependent and dynamic aspects in terms of wetting rate, it was found that the proposed dynamic pollution model results in more detailed and realistic field profiles around insulators than those obtained with a constant value of conductivity.

## Figures and Tables

**Table 1:** Reference leakage current and surface conductance.

Parameter	Ins. 1	Ins. 2	Ins. 3	Ins. 4	Ins. 5	Standard Deviation
Leakage current (mA)	0.195	0.190	0.187	0.191	0.185	0.0039
conductance (µS)	3.882	3.782	3.723	3.802	3.683	0.0765

Ins. = Insulator.

## Data Availability

Data used in this research has been provided in the paper.
